# Mitochondrial Reactive Oxygen Species Regulate Immune Responses of Macrophages to *Aspergillus fumigatus*

**DOI:** 10.3389/fimmu.2021.641495

**Published:** 2021-03-25

**Authors:** Remi Hatinguais, Arnab Pradhan, Gordon D. Brown, Alistair J. P. Brown, Adilia Warris, Elena Shekhova

**Affiliations:** Medical Research Council Centre for Medical Mycology at the University of Exeter, Geoffrey Pope Building, University of Exeter, Exeter, United Kingdom

**Keywords:** macrophages, reverse electron transport, reactive oxygen species, mitochondria, *Aspegillus fumigatus*, cytokines

## Abstract

Reactive Oxygen Species (ROS) are highly reactive molecules that can induce oxidative stress. For instance, the oxidative burst of immune cells is well known for its ability to inhibit the growth of invading pathogens. However, ROS also mediate redox signalling, which is important for the regulation of antimicrobial immunity. Here, we report a crucial role of mitochondrial ROS (mitoROS) in antifungal responses of macrophages. We show that mitoROS production rises in murine macrophages exposed to swollen conidia of the fungal pathogen *Aspergillus fumigatus* compared to untreated macrophages, or those treated with resting conidia. Furthermore, the exposure of macrophages to swollen conidia increases the activity of complex II of the respiratory chain and raises mitochondrial membrane potential. These alterations in mitochondria of infected macrophages suggest that mitoROS are produced *via* reverse electron transport (RET). Significantly, preventing mitoROS generation *via* RET by treatment with rotenone, or a suppressor of site IQ electron leak, S1QEL1.1, lowers the production of pro-inflammatory cytokines TNF-α and IL-1β in macrophages exposed to swollen conidia of *A. fumigatus*. Rotenone and S1QEL1.1 also reduces the fungicidal activity of macrophages against swollen conidia. Moreover, we have established that elevated recruitment of NADPH oxidase 2 (NOX2, also called gp91phox) to the phagosomal membrane occurs prior to the increase in mitoROS generation. Using macrophages from gp91phox^-/-^ mice, we have further demonstrated that NOX2 is required to regulate cytokine secretion by RET-associated mitoROS in response to infection with swollen conidia. Taken together, these observations demonstrate the importance of RET-mediated mitoROS production in macrophages infected with *A. fumigatus*.

## Introduction

*Aspergillus fumigatus* is a ubiquitous fungus that causes a wide range of illnesses, from allergic reactions to life-threatening invasive aspergillosis (1). Primary immunodeficiency is one of the conditions that places individuals at risk of deadly invasive aspergillosis (2). For instance, patients with chronic granulomatous disease (CGD), who have mutations in genes encoding the nicotinamide adenine dinucleotide phosphate (NADPH) oxidase complex, are highly susceptible to bacterial and fungal infections including invasive aspergillosis (3, 4). The lack of NADPH oxidase (NOX)-derived reactive oxygen species (ROS) in CGD phagocytes results in the defective oxidative killing of pathogens (3).

Besides being crucial for intracellular oxidative killing, ROS can also orchestrate inflammatory processes in response to microbial pathogens including fungi (5–7). Changes in ROS levels are often a consequence of metabolic remodelling in activated immune cells. Together these changes in cellular metabolism and redox homeostasis can initiate, as well as resolve, inflammatory responses (8–12). Indeed, a growing number of investigations have shown that tuning the production of specific inflammatory mediators, cytokines, correlates directly with metabolic re-programming and altered ROS production (9, 11).

There are numerous potential cellular sources of ROS, but the mitochondrial respiratory chain complex I is known to be one of the main contributors (13). ROS can be generated at complex I *via* a specific mechanism called reverse electron transport (RET). RET occurs when a significantly reduced coenzyme Q pool and a large pH gradient across the mitochondrial inner membrane drive electrons backwards through complex I resulting in elevated superoxide production (14). Therefore, two types of metabolic alterations can lead to RET. First, RET may occur when the coenzyme Q pool becomes over-reduced with electrons from respiratory chain complex II or glycerol-3-phosphate dehydrogenase (9, 11, 14, 15). Second, a reduction in ATP production by oxidative phosphorylation may promote the increased membrane potential needed to sustain RET (9, 14).

The process of mitochondrial ROS (mitoROS) generation *via* RET has been known for decades, but its physiological relevance has been shown only recently (15). A growing body of evidence indicates that RET occurs under physiological conditions and that it drives redox signalling in a variety of processes *in vivo*. Specific targets of RET-generated mitoROS remain unidentified, but may include redox-sensitive proteins that are modified (and thus inhibited or activated) by mitoROS. Nevertheless, the generation of mitoROS by RET has been shown to be crucial for survival under stress (16), oxygen sensing by the carotid body (17), and control of inflammation (9, 11). In addition, although high levels of ROS can promote aging by causing oxidative damage to cellular components, increasing mitoROS production specifically *via* RET extends *Drosophila* lifespan (18). Therefore, identifying the mechanisms that trigger mitoROS generation *via* RET and defining their targets will improve our understanding of physiological and pathophysiological signalling transduction, and may suggest new avenues for therapeutic manipulation.

Previously, it was shown that macrophages stimulated by the bacterial product lipopolysaccharide (LPS) shift their metabolism from oxidative phosphorylation towards glycolysis, which has also been associated with an increase in RET-induced mitoROS production. In turn, mitoROS promote stabilization of the transcription factor, hypoxia-inducible factor 1α, resulting in elevation of interleukin (IL)-1β expression (9). It was recently demonstrated that infection with *A. fumigatus* enhances glycolysis in macrophages, which is required for efficient innate immune responses (19). In this study, we have shown that mitoROS are produced *via* RET in macrophages infected with swollen *A. fumigatus* conidia and that this contributes to redox signalling necessary for cytokine secretion and fungal growth inhibition. In particular, we have found that blocking RET with a mitochondrial inhibitor, rotenone, or an antioxidant, S1QEL1.1, prevents tumor necrosis factor-α (TNF-α) and IL-1β secretion in murine macrophages exposed to swollen fungal conidia. Furthermore, both rotenone and S1QEL1.1 abolish the ability of macrophages to inhibit growth of swollen conidia, while having no effect on their ability to inactivate resting conidia. Moreover, we show that enhanced recruitment of NOX2 (gp91phox) to phagosomes occurs before the increase in mitoROS generation by infected macrophages. Using macrophages from gp91phox^-/-^ mice, we have further established that NOX2 activity is essential for the regulation of cytokine secretion *via* RET-derived mitoROS. Overall, our work reveals a novel mechanism underlying the regulation of antifungal responses of macrophages against *Aspergillus* infection.

## Materials and Methods

### Reagents

Red Mitochondrial Superoxide Indicator (MitoSOX) (M36008), Pierce Trifluoroacetic Acid (TFA) (28904), dithiothreitol (DTT) (R0862), zymosan (Z2849) were obtained from Thermo Fisher Scientific. RPMI 1640 Medium, GlutaMAX (61870036), heat inactivated Fetal Bovine Serum (HI-FBS) (10082147), HEPES (1156049), EDTA (15575020), Phosphate-Buffered Saline (PBS) (10010023), HBSS (14025-092) were purchased from Gibco. Triton X-100 (X100), diamide (D3648), Triethylammonium bicarbonate buffer (TEAB) (T7408), NaCl (S9888), iodoacetamide (I1149), SDS (L3771), Tris(2-carboxyethyl)phosphine hydrochloride (TCEP) (C4706), urea (U5378), acetonitrile (ACN) (271004), HEPES (H4034), rotenone (R8875), mitoTEMPO (SML0737), S1QEL1.1 (SML1948), S3QEL2 (SML1554) were purchased from Sigma-Aldrich. Thiopropyl Sepharose 6B resin (17042001) was obtained from GE Healthcare.

### Cells and Preparation of *A. fumigatus*

Murine bone marrow-derived macrophages (BMDMs) were obtained from C57BL/6J or gp91phox^−/−^mice, as described previously (20). Cells were differentiated in RPMI 1640 Medium, GlutaMAX supplemented with 20% (v/v) L929 cell supernatant, 10% (v/v) HI-FBS, 100 µg/ml penicillin, 100 µg/ml streptomycin, and 10 mM HEPES. At least one night before treatment, BMDMs were transferred into RPMI 1640 Medium, GlutaMAX supplemented with 10% (v/v) HI-FBS (R10 medium). Alveolar macrophages were isolated from murine bronchoalveolar lavage fluid obtained by washing the airways with sterile PBS containing 5mM EDTA (21). Murine peritoneal macrophages were harvested 4 days after intraperitoneal injection of 3% (w/v) thioglycollate (BD Biosciences) by washing the intraperitoneal cavity with sterile PBS containing 5mM EDTA (20). Isolated cells were plated in multi-well TC culture plates in R10 medium and left to adhere for at least 4 h with a subsequent washing off non-adherent cells.

*A. fumigatus* clinical isolate CBS 144-89 (CEA10) was used as wild-type strain (22). To test a DHN melanin-deficient strain, Δ*alb1*(*pksP1*) was used (23). To prepare swollen conidia of *A. fumigatus*, conidia were first grown in Sabouraud-dextrose media for 3 h at 30 rpm to avoid clumping. Swollen conidia were harvested, washed and re-suspended in R10 medium. To fix swollen conidia, after swelling, they were treated with 10% (v/v) formalin for 1 h at 4°C, subsequently washed with PBS and re-suspended in R10 medium. To prepare heat-killed conidia, a suspension of conidia in water were incubated at 90°C for 30 min.

### Measurement of Mitochondrial Reactive Oxygen Species and NOX2 Localization

For fluorescence microscopy, 4x10^4^ BMDMs in R10 medium were inoculated into µ-Slide 8-well coverslip (ibidi) and incubated at 37°C in 5% (v/v) CO_2_. Cells were then infected with *A. fumigatus* or exposed to zymosan. 20 min before the end of incubation, cells were stained with 5 μM MitoSOX in HBSS, and then fixed with 10% (v/v) formalin. To evaluate NOX2 cellular localization, BMDMs were exposed to conidia, methanol fixed and stained with gp91-phox Alexa Fluor 647 antibody (Santa Cruz, sc-130543 AF647). Fluorescence was measured using a DeltaVision fluorescent microscope. Images were analyzed using ImageJ software and measuring the corrected total cell fluorescence (CTCF). CTCF was calculated according to the formula: CTCF = Integrated Density - (Area of selected cell x Mean fluorescence of background readings). Brightness and contrast adjustments are the same for all representative images.

### Measurement of Mitochondrial Membrane Potential

The mitochondrial transmembrane potential (ΔΨm) was assessed by the measurement of uptake of MitoView633 (Cambridge Bioscience, BT70055) fluorescent probe that was monitored fluorimetrically in a microplate reader (Tecan, SPARK1933) with a far-red channel.

### Cysteine Oxidation

BMDMs at a concentration of 2 × 10^6^ cells/well in TC-treated six-well cell culture plates were challenged with swollen *A. fumigatus* conidia (MOI 5) for 2 h. After incubation, macrophages were washed with PBS and samples were pre-processed for an analysis of oxidative protein modifications as described previously (24). Briefly, proteins were precipitated with TCA, and protein pellets were dissolved in 300 µl lysis buffer containing 1% (w/v) SDS, 150 mM NaCl, 100 mM TEAB, 4 mM EDTA, 1 tablet of cOmplete Ultra Protease Inhibitor Cocktail (Roche), and 100 mM iodoacetamide (IAM). After excess IAM was removed with acetone, proteins were re-solubilized in 100 mM TEAB and reduced by the addition of 0.5 M TCEP (tris(2-carboxyethyl)phosphine). Reduced proteins were captured on a pre-conditioned Thiopropyl Sepharose 6B resin. After incubation on the resin, unbound proteins were washed away with the washing buffers in the following order: 8 M urea; 2 M NaCl; 80% (v/v) ACN and 0.1% (v/v) TFA; and 25 mM HEPES. To elute cysteine-containing proteins, resin was incubated with 25 mM NH_4_HCO_3_ buffer containing 20 mM DTT and centrifuged at 1,500g for 1 min. Fractions were analyzed by SDS-gel electrophoresis. Protein concentration in each sample was determined by Bradford assay (25). Equal amounts of proteins were loaded and separated on NuPAGE 4–12% Bis-Tris Gel (Invitrogen) and gels were stained by silver staining kit (Thermo Scientific, 24612). SeeBlue Plus2 (Invitrogen, LC5925) was used as a marker. Western blot analysis was performed to evaluate the level of thiol modifications in GAPDH. MagicMark XP (Invitrogen, LC5602) was used as marker for western blots.

### Mitochondrial Respiratory Chain Assays and Evaluation of Protein Expression Levels

BMDMs were plated at 2 × 10^6^ cells/well in non-treated six-well cell culture plates, challenged with *A. fumigatus* at MOI of 5 and plates were subsequently spun at 400g for 2 min. After incubation for 2 h, cells were washed with PBS and put on ice. Cells were harvested and centrifuged at 1000 g for 10 min at 4°C and frozen in liquid nitrogen. After cells were quickly thawed at 37°C, samples were dissolved in PBS containing cOmplete Ultra Protease Inhibitor Cocktail (Roche) and homogenized with a 50-μl Hamilton syringe (Sigma, 58382) by taking up and expelling the suspension several times until it appeared as a homogeneous solution. Homogenate was kept on ice for the immediate measurement enzymatic activities. Enzymatic activity of respiratory chain complexes was performed as previously described with a spectrophotometer Spark plate reader (26). Cell homogenates were also used to evaluate the abundance of proteins in BMDMs treated with swollen conidia of *A. fumigatus.* Extracted proteins were analyzed by western blot with anti-GAPDH (Abcam, ab181602), anti-GLRX1 (R&DSystems, AF3119), anti-SOD2 (Abcam, ab13533) or NOX2 (Abcam, ab129068) antibodies.

### Cytokine and Killing Assays

Macrophages were plated at 4 × 10^5^ cells/well in TC-treated 48-well cell culture plates and left to adhere overnight. Cells were subsequently incubated with swollen *A. fumigatus* conidia (MOI 5). Zymosan (MOI 5) was given where indicated. 2 µM diphenyleneiodonium chloride (DPI) was added for 1 h to inhibit NADPH oxidase complex. 2.5 µM rotenone or 12 µM FCCP were added 1 h before challenge. 0.5 mM MitoTEMPO, 5 µM S1QEL1.1, or 5 µM S3QEL2 were added to macrophages for the whole period of incubation with swollen conidia. Supernatants from macrophages were collected at 14 h (overnight) after stimulation. IL-1β (DY401) and TNF-α (Dy410) ELISA kits from R&DSystems were used according to the manufacturer’s instructions. To assess killing capacity, macrophages were plated at 5 × 10^4^ cells/well in TC-treated 96-well cell plates and incubated with conidia at MOI of 3 for resting conidia and MOI of 1 for swollen conidia. Following incubation for 4 hours, cells were lysed and the viability of remaining conidia was determined with resazurin assay (27).

### Statistical Analyses

RStudio was utilized to perform data analyses and visualization. The following packages were used: base (28), car (29), data.table (30), ggplot2 (31), ggpubr (32), rstatix (33), plyr (34), carData (35). To test whether obtained data were normally distributed the Shapiro-Wilk test was performed. Levene’s test was used to assess homogeneity of variance. Statistical significance was evaluated either with the unpaired standard Student’s or Welch’s *t*-test or Mann Whitney U Test. The false discovery rate (FDR) adjustment of the p values was implemented using the Benjamini-Hochberg method (36). Multiple groups were compared either with the Kruskal-Wallis test or Welch’s one-way ANOVA or one-way ANOVA, all followed by Tukey post-hoc tests. * indicates p<0.05, ** indicates p<0.01, *** indicates p<0.001.

## Results

### Sensing Swollen *A. fumigatus* Conidia Induces MitoROS in Macrophages

**Figure 1:**
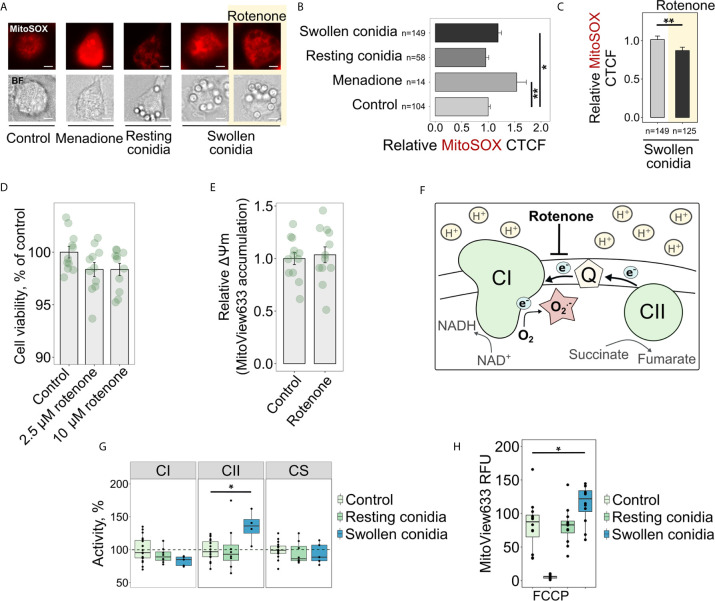
Sensing swollen conidia of *A. fumigatus* induces mitoROS production and increases the activity of the respiratory chain complex II and mitochondrial transmembrane potential. **(A)** Microscopic analysis of mitoROS in BMDMs infected with resting or swollen conidia of *A. fumigatus.* BMDMs were incubated with either resting or swollen *A. fumigatus* conidia for 2 h, stained with mitoSOX, fixed and subjected to imaging. Treatment with menadione was used as a positive control. To prevent mitoROS production through RET, BMDMs were treated with rotenone (shown with yellow background) for 1 h prior to exposure of BMDMs to swollen conidia. The scale bars are 5 µm. **(B, C)** The quantification of the level of fluorescence in infected macrophages. The abundance of mitoROS in BMDMs was determined by image analysis of MitoSOX-stained BMDMs with ImageJ software. A relative corrected total cell fluorescence (CTCF) was calculated in relation to an average of CTCF measured in untreated (Control) BMDMs **(B)** or in BMDMs exposed to swollen conidia **(C)**. **(D)** Measurement of viability of BMDMs after a short-term treatment with rotenone. Viability of BMDMs measured with the resazurin assay after 1 h of exposure to rotenone followed by 2 h cultivation in R10 media and is expressed in comparison to untreated cells (Control). **(E)** Analysis of mitochondrial membrane potential after treatment of BMDMs with rotenone. MitoView633-associated fluorescence was measured in BMDMs following 1 h of exposure to 2.5 µM rotenone followed by 2 h cultivation in R10 media containing fluorescent probe and represented mitochondrial membrane potential. Relative mitochondrial membrane potential was calculated in relation to an average of fluorescence units measured in untreated (Control) cells. **(F)** Schematic of mitoROS production *via* reverse electron transport (RET) in mitochondria. Coenzyme Q (Q) becomes over-reduced with electrons supplied by complex II (CII) during succinate oxidation. A large membrane potential and pH gradient drive electrons from coenzyme Q to complex I (CI) resulting in superoxide (O_2_^.-^) production at one of CI sites. Rotenone inhibits RET-induced mitoROS generation. **(G)** Spectrophotometry analysis of enzyme activities of mitochondrial respiratory chain complex I (CI), complex II (CII), and citrate synthase (CS). BMDMs were left untreated (Control) or exposed to resting or swollen conidia of *A. fumigatus* for 2 h. Enzymatic activity measured in untreated BMDMs was set as 100% in each replicate. The activity of citrate synthase was measured to assess quality of mitochondria. **(H)** Measurements of mitochondrial membrane potential in BMDMs by detecting fluorescence of MitoView633. BMDMs were exposed to heat-inactivated resting or formalin-fixed swollen conidia in the presence of MitoView633. MitoView633-associated fluorescence was measured after incubation overnight. Treatment with 18 µM FCCP (p-trifluoromethoxyphenylhydrazone) was used as a negative control. Data are from two **(D, E, H)**, three **(B, C)**, or four **(G)** independent experiments. “n” indicates number of cells used for quantification; bars indicate means and standard errors. Statistical significance was calculated with the Kruskal-Wallis test followed by Tukey post-hoc tests **(B, H)**, with the Mann Whitney *U* Test **(C)**, or Student’s **(D, E)** *t*-test, or one-way ANOVA followed by Tukey post-hoc tests **(G)**: * indicates p<0.05, and ** indicates p<0.01.

Recently, it was proposed that mitoROS, produced specifically through RET, transform activated macrophages into a pro-inflammatory state (9). However, it was not known whether the recognition of fungal cells, or fungal cell wall components, induces the increased mitoROS production in macrophages. Therefore, our first objective was to test whether macrophages infected with the fungal pathogen *A. fumigatus* display increased mitoROS generation. Interestingly, using mitoSOX as a fluorescent probe for mitoROS, we observed that resting conidia of *A. fumigatus* did not impact mitoROS production by BMDMs after a short-term co-incubation of 2 h (**Figures 1A, B**). In contrast to resting conidia, infection with germinating (swollen) conidia of the fungus for the same period led to elevated mitoROS production in macrophages (**Figures 1A, B**). The mitochondrial inhibitor rotenone is known to prevent mitoROS generation during RET (13, 37, 38) (**Figure 1F**), and therefore we tested the effect of treating BMDMs with rotenone before exposure to swollen conidia. Pre-treatment with rotenone for 1 h significantly inhibited the increase in mitoROS normally observed following exposure of BMDMs to swollen *A. fumigatus* conidia (**Figures 1A, C**). This was consistent with our prediction that *A. fumigatus* triggers elevated mitoROS generation in macrophages *via* RET.

As rotenone is a metabolic inhibitor, and therefore its potential toxicity could conceivably have accounted for the observed reduction in mitoROS production in infected macrophages. However, the rotenone concentration used (2.5 µM) did not alter viability of macrophages (**Figure 1D**). Even a 4-fold increase in rotenone concentration did not affect metabolic activity of BMDMs after a 1 h exposure (**Figure 1D**). In addition, mitochondrial membrane potential was not affected by rotenone (**Figure 1E**), suggesting that a short-term pre-treatment of BMDMs with 2.5 µM rotenone did not interfere with a mitochondrial potential-dependent accumulation of probes such as MitoSOX. These observations are consistent with a previous report about the dose- and time-dependent toxicity of rotenone (39). Therefore, the impact of this short-term pre-treatment of BMDMs with 2.5 µM rotenone supports the idea that *A. fumigatus* activates RET-mediated mitoROS production.

RET is characterized by a considerably reduced coenzyme Q pool and a subsequent transfer of electrons from coenzyme Q to complex I, and the increased activity of mitochondrial complex II can contribute to the excessive reduction of the coenzyme Q pool (**Figure 1F**). Therefore, we hypothesized that the elevated activity of complex II could facilitate mitoROS production *via* RET in fungi-challenged macrophages. As predicted, we found that the activity of complex II increased when BMDMs were treated with swollen conidia of *A. fumigatus*, compared to unstimulated BMDMs (**Figure 1G**). Furthermore, the activity of complex II was not affected by resting conidia, which were consistently unable to induce elevated mitoROS levels (**Figures 1A, B**). These data support the idea that complex II drives mitoROS production *via* RET.

Mitochondrial complex I is the site for mitoROS production *via* RET (**Figure 1F**), and the activity of this complex can be decreased due to inhibition by mitoROS produced at that site. Thus, the reduced activity of this complex is another indicator of RET taking place (38). Interestingly, when we measured the activity of complex I in infected cells, we could only detect a slight reduction in the complex I activity that was not statistically significant in BMDMs exposed to swollen conidia (**Figure 1G**).

Increased mitochondrial membrane potential, which occurs due to a reduction in ATP production by oxidative phosphorylation, can contribute to and help to sustain RET. We used MitoView633 to monitor mitochondrial membrane potential in infected BMDMs, as the mitochondrial accumulation of MitoView633 and its subsequent fluorescence are dependent on the membrane potential. To prevent overgrowth of the fungus during overnight co-incubation with BMDMs, we used inactivated conidia in this experiment. Consistent with our previous results, the mitochondrial membrane potential was increased in BMDMs infected with swollen conidia, but not following exposure to resting conidia (**Figure 1H**).

Next, as *A. fumigatus* infection is associated with increased mitoROS (**Figures 1A–C**), we tested whether infection affects the redox status of endogenous BMDM proteins. To achieve this, we assessed the levels of reversibly oxidized proteins in macrophages infected with swollen conidia of *A. fumigatus*. Proteins were extracted from BMDMs and pre-processed according to the scheme in **Supplemental Figure 1A**. This method allowed us to capture proteins containing oxidized cysteines, which are the primary targets of ROS. This analysis of thiol oxidation showed that exposing macrophages to the fungus promoted oxidative post-translation modifications in the immune cells (**Supplementary Figure 1B**). We also tested whether the high mitoROS in fungus-activated BMDMs induces oxidative stress by evaluating the abundance of two antioxidant proteins: cytosolic glutaredoxin 1 (GLRX1) and mitochondrial superoxide dismutase (SOD2). The expression of neither protein was increased following exposure to swollen *A. fumigatus* conidia, as measured by western blotting (**Supplementary Figures 1C, D**) suggesting no significant exposure to oxidative stress.

### Removal of a Melanin or Protein Layer From Dormant *A. fumigatus* Conidia Does Not Induce MitoROS in Infected Macrophages

**Figure 2:**
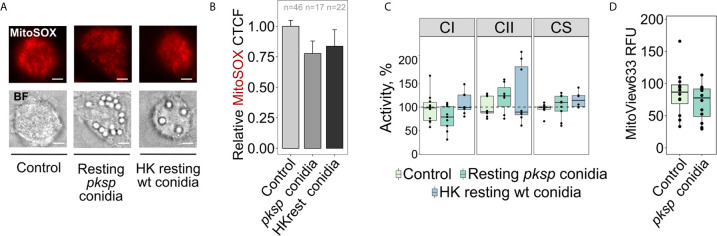
Detection of melanin-deficient or heat-killed resting *A. fumigatus* conidia by BMDMs does not induce mitoROS or alter the activity of mitochondrial complexes I and II, and mitochondrial transmembrane potential. **(A)** Microscopic analysis of mitoROS in BMDMs. BMDMs were left untreated (Control) or infected with conidia lacking DHN melanin (*pksp*) or heat-killed (HK) resting *A. fumigatus* conidia for 2 h. Cells were stained with mitoSOX, fixed and subjected to imaging. The scale bars are 5 µm. **(B)** The abundance of mitoROS in BMDMs was determined by image analysis of MitoSOX-stained BMDMs with ImageJ software. A relative corrected total cell fluorescence (CTCF) was calculated in relation to an average of CTCF measured in untreated (Control) BMDMs. **(C)** Spectrophotometry analysis of enzyme activities of mitochondrial respiratory chain complex I (CI), complex II (CII), and citrate synthase (CS). BMDMs were left untreated (Control) or exposed to resting or heat-killed resting conidia for 2 h. Enzymatic activity measured in untreated BMDMs was set as 100% in each replicate. The activity of citrate synthase was measured to assess quality of mitochondria. **(D)** Measurements of mitochondrial membrane potential in BMDMs by detecting fluorescence of MitoView633. BMDMs were exposed to *pksp* resting conidia. MitoView633 fluorescence was measured after incubation overnight. Data are from two **(B, D)**, or three **(C)** independent experiments. “n” indicates number of cells used for quantification; bars indicate means and standard errors. Differences between groups were evaluated with the one-way ANOVA.

We speculated that DHN melanin on the surface of dormant *A. fumigatus* could prevent recognition of immunogenic components of the fungal surface required to initiate RET. Therefore, to investigate why resting conidia do not induce mitoROS *via* RET in macrophages, we exposed BMDMs to resting conidia of a *A. fumigatus* Δ*pksP* strain, which lacks the DHN melanin layer (23). Interestingly, we observed neither an increase in mitoROS levels (**Figures 2A, B**) nor a significant alteration in the activities of complex I or complex II, or in mitochondrial membrane potential in macrophages infected with this DHN melanin-deficient strain (**Figures 2C, D**).

Next, we tested whether proteins on the conidial surface could block the sensing of immunogenic fungal molecules that initiate mitoROS production *via* RET (40). Surface proteins were degraded by heating and heat-treated resting conidia were added to BMDMs. However, no changes in mitoROS production (**Figures 2A, B**) or in the mitochondrial activities and membrane potential were observed in macrophages following incubation with heat-killed resting conidia (**Figure 2C**, and **Figure 1H**). Overall, these data suggest that only germinating (swollen) *A. fumigatus* conidia are able to induce RET.

### Inhibition of RET in Macrophages Exposed to Swollen *A. fumigatus* Conidia Reduces Cytokine Secretion and Fungicidal Capacity

**Figure 3:**
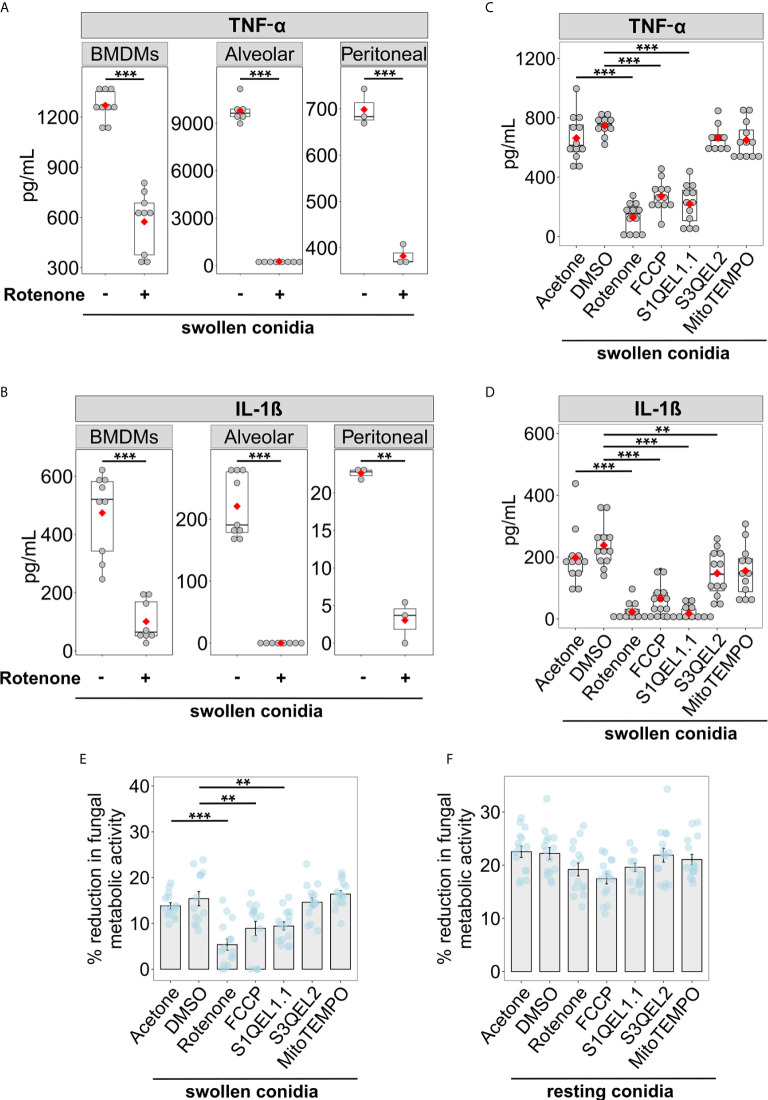
Suppression of RET-induced mitoROS reduces cytokine secretion and the ability to inhibit growth of swollen *A. fumigatus* conidia by murine macrophages. **(A, B)** Evaluation of cytokine secretion by infected macrophages. Bone marrow-derived macrophages (BMDMs), alveolar or peritoneal macrophages were treated either with vehicle (acetone) or rotenone for 1 h, after washing, macrophages were exposed to swollen *A. fumigatus* conidia and the supernatant was collected after incubation overnight. Levels of TNF-α **(A)** and IL-1β **(B)** in supernatants were analyzed by ELISA. **(C, D)** BMDMs were ether treated with vehicle (acetone or DMSO), rotenone, or FCCP for 1 h. After compounds were removed, BMDMs were treated with swollen *A. fumigatus* conidia. Antioxidants S1QEL1.1, S3QEL2, or mitoTEMPO were added together with conidia. Following incubation overnight, supernatants were collected to measure cytokine levels by ELISA. No cytokines were detected in the supernatant from cells treated with tested compounds, but not treated with swollen conidia (not shown). **(E, F)** BMDMs were ether treated with vehicle (acetone or DMSO), rotenone, or FCCP for 1 h. After compounds were removed, BMDMs were treated with swollen or resting *A. fumigatus* conidia. Antioxidants S1QEL1.1, S3QEL2, or mitoTEMPO were added together with conidia where indicated. After incubation for 4 h, macrophages were lysed with water containing Triton X-100, and the growth of *A. fumigatus* was measure by a metabolic activity assay based on resazurin. The metabolic activity of *A. fumigatus* conidia that were not exposed to BMDMs was set to 100%. Data are from two **(A, B)** (except for values for peritoneal macrophages, that were obtain from one experiment), or three **(C–F)** independent experiments. Bars indicate means and standard errors, red rhomb represent means. Statistical significance was calculated with the Welch’s *t*-test **(A, B)**, one-way ANOVA **(C–E)** or Welch’s one-way ANOVA **(F)** followed by Tukey post-hoc tests: ** indicates p<0.01, and *** indicates p<0.001.

The secretion of pro-inflammatory cytokines such as TNF-α and IL-1β helps to develop host resistance to *A. fumigatus* infections and mediate antifungal immune responses (41, 42). Therefore, we tested whether blocking RET-mediated mitoROS production affects the ability of macrophages to secrete these cytokines in response to fungal invasion. Murine macrophages were pre-treated with rotenone for 1 h and exposed to swollen *A. fumigatus* conidia. We confirmed the importance of mitoROS for antifungal responses in bone marrow derived, alveolar, and peritoneal macrophages, as blocking mitoROS with rotenone resulted in reduced secretion of TNF-α and IL-1β by all three types of macrophages infected with swollen conidia (**Figures 3A, B**). This suggests a universal function of RET-associated mitoROS signalling in the antifungal responses of macrophages.

To confirm that the effect of rotenone is primarily due to its ability to suppress mitoROS production *via* RET, we tested whether other inhibitors of RET affect cytokine secretion in *A. fumigatus*-stimulated BMDMs. Accordingly, carbonyl cyanide p-trifluoromethoxyphenylhydrazone (FCCP), which is known to prevent RET by altering mitochondrial membrane potential (14), significantly reduced cytokine secretion in infected BMDMs (**Figures 3C, D**). Also, S1QEL1.1, an antioxidant that suppresses mitoROS *via* RET (43), decreased TNF-α and IL-1β production by BMDMs exposed to swollen conidia. In contrast, other mitochondrial antioxidants, S3QEL2 and mitoTEMPO, which act independently of RET suppression (44), did not affect TNF-α secretion by BMDMs exposed to swollen conidia (**Figures 3C, D**). Taken together, these observations strongly suggest that preventing RET-mediated mitoROS generation impairs the cytokine responses of macrophages.

Next, we tested whether mitoROS production is necessary for macrophages to inhibit *A. fumigatus* growth. Interestingly, compounds with potential to suppress RET, namely rotenone, FCCP and S1QEL1.1, reduced the capacity of macrophages to inhibit growth of swollen conidia (**Figure 3E**). In contrast, the antioxidants S3QEL2 and mitoTEMPO, which do not affect RET, did not alter the fungicidal function of BMDMs against swollen conidia (**Figure 3E**). Interestingly, the RET inhibitors did not attenuate the inhibitory capacity of macrophages against resting conidia (**Figure 3F**). This is entirely consistent with our observation that resting conidia do not induce RET-mediated mitoROS generation (**Figures 1A, B, G, H**). Therefore, mitoROS that originate specifically from RET mediate cytokine secretion and are required to inhibit growth of swollen conidia, but are dispensable for the fungicidal function against resting conidia.

### Sensing β-Glucan or Viability-Associated Molecules Does Not Induce MitoROS *via* RET

**Figure 4:**
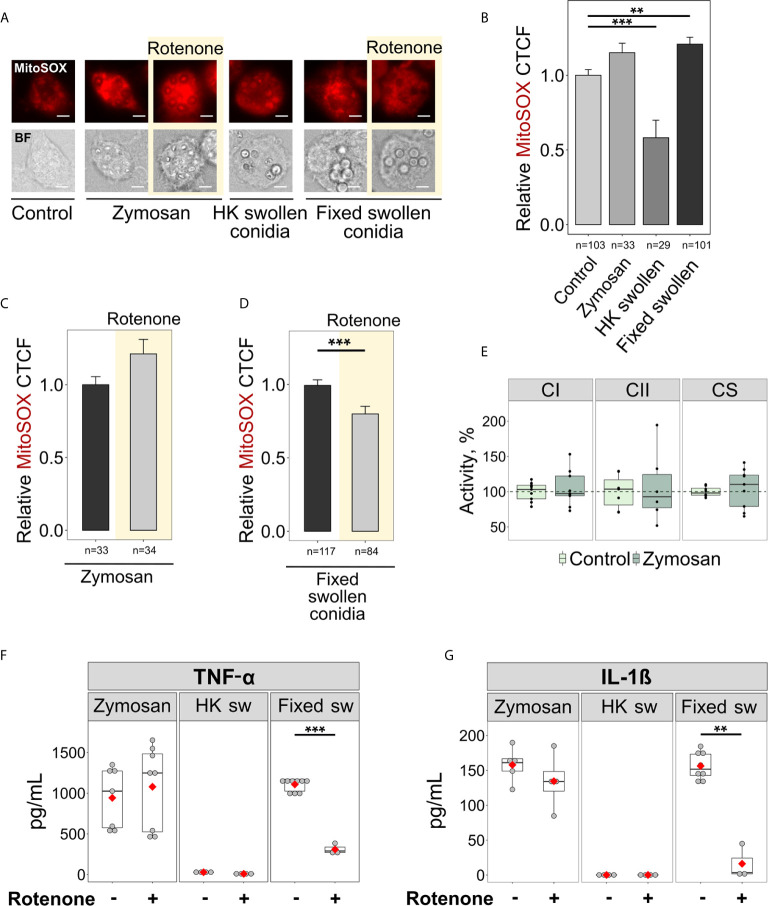
Sensing fixed swollen *A. fumigatus* conidia but not zymosan or heat-killed swollen conidia induces mitoROS production *via* RET in BMDMs. **(A)** Microscopic analysis of mitoROS in BMDMs. BMDMs were left untreated (Control) or exposed to zymosan particles, heat-killed (HK) or formalin-fixed swollen conidia of *A. fumigatus*. Cells were stained with mitoSOX, fixed and subjected to imaging. Where indicated, BMDMs were pre-treated with rotenone (shown with yellow background) for 1 h. The scale bars are 5 µm. **(B–D)** The abundance of mitoROS in BMDMs was determined by image analysis of MitoSOX-stained BMDMs with ImageJ software. A relative corrected total cell fluorescence (CTCF) was calculated in relation to an average of CTCF measured in untreated (Control) BMDMs **(B)** or in BMDMs exposed to zymosan **(C)** or formalin-fixed swollen conidia **(D)**. **(E)** Spectrophotometry analysis of enzyme activities of mitochondrial respiratory chain complex I (CI), complex II (CII), and citrate synthase (CS). Enzymatic activity measured in untreated BMDMs were set as 100% in each replicate. The activity of citrate synthase was measured to assess quality of mitochondria. **(F, G)** Evaluation of cytokine secretion in stimulated macrophages. After treatment with either vehicle or rotenone, BMDMs were exposed to zymosan particles, heat-killed (HK sw) or formalin-fixed (Fixed sw) swollen conidia of *A. fumigatus*. Levels of TNF-α and IL-1β in supernatants were analyzed by ELISA. Data are from two **(B–D, F, G)**, or three **(E)** independent experiments. “n” indicates number of cells used for quantification; bars indicate means and standard errors, red rhomb represent means. Statistical significance was calculated with the Kruskal-Wallis test followed by Tukey post-hoc test **(B)**, Mann Whitney U Test **(C, D)**, or Student’s *t*-test **(E–G)**: ** indicates p<0.01, and *** indicates p<0.001.

The recognition of microbial ligands by various receptors is required to initiate immune responses and antimicrobial signalling (45). Currently, little is known about the ligands that induce inflammatory responses through RET-associated mitoROS signalling. Since we only observed RET and elevated mitoROS generation in BMDMs infected with swollen conidia, we reasoned that RET might be triggered by β-glucan, a major carbohydrate of the cell wall of swollen *A. fumigatus* conidia. Interestingly, macrophages activated by zymosan particles, a fungal cell wall-derived product composed mainly of β-glucan, exhibited elevated mitoROS generation as detected by mitoSOX staining (**Figures 4A, B**). However, the increase in mitoROS was not statistically significant and was not inhibited by rotenone (**Figures 4A–C**), suggesting that the mitoROS observed upon zymosan stimulation was not generated *via* RET. This correlated well with the observation that zymosan was unable to elevate complex II activity required for RET (**Figure 4E**) and that rotenone had no effect on TNF-α and IL-1β secretion when BMDMs were activated by zymosan (**Figures 4F, G**). Therefore, β-glucan recognition is not sufficient to trigger RET-mediated mitoROS production.

The metabolic reprogramming of macrophages, which could lead to mitoROS production *via* RET, takes place when immune cells are infected with live *E.coli*, whereas heat-killed bacteria do not have this effect upon macrophage metabolism (10). This may indicate that viability-associated molecules like microbial mRNA can induce metabolic reprogramming required for RET in immune cells (46). Thus, we also tested whether this “viability-specific” immune response triggers mitoROS in *A. fumigatus*-infected macrophages. Surprisingly, while heat-killed swollen conidia did not induce mitoROS, and even reduced their levels, infection with formalin-fixed swollen conidia led to an increase in mitoROS generation in BMDMs (**Figures 4A, B**). This elevation in mitoROS was abolished upon pre-treatment with rotenone, confirming that fixed germinating conidia can induce RET-associated mitoROS (**Figure 4D**). Furthermore, rotenone treatment reduced the production of TNF-α and IL-1β by macrophages challenged with fixed swollen conidia (**Figures 4F, G**). Meanwhile, heat-killed swollen conidia did not stimulate cytokine secretion by macrophages, which was consistent with their inability to initiate mitochondria redox signalling (**Figures 4F, G**). These data indicate that the regulation of cytokine production by RET does not depend upon sensing viability-associated molecules. Therefore, recognition of the cell surface of swollen conidia, is probably required for the induction of RET-mediated mitoROS production.

### NADPH Oxidase 2 Is Required for MitoROS Production *via* RET in *A. fumigatus*-Stimulated Macrophages

**Figure 5:**
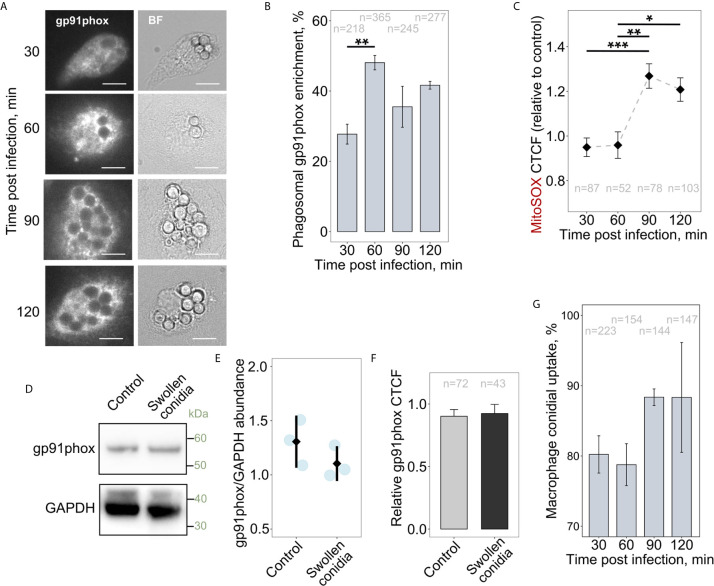
Recruitment of gp91phox to phagosomes occurs prior to the increase in mitoROS production. **(A)** Microscopic analysis of gp91phox localization in BMDMs. Cells were treated with swollen *A. fumigatus* conidia for an indicated period of time, fixed, stained with gp91phox antibodies, and subjected to imaging. The scale bars are 10 µm. **(B)** Quantification of the phagosomal enrichment of gp91phox in BMDMs following infection with swollen conidia. **(C)** The abundance of mitoROS in BMDMs was determined by image analysis of MitoSOX-stained BMDMs with ImageJ software. Corrected total cell fluorescence (CTCF) in infected cells was normalized to an average of CTCF measured in untreated (control) cells. Means of CTCF for each time point were compared. **(D)** Western blot analysis of protein abundance in untreated (Control) and infected for 2 h with swollen conidia BMDMs. Proteins were extracted from macrophages and analyzed by western blot with anti-gp91phox or GAPDH antibodies. **(E)** Quantification of intensity of corresponding bands on blots as shown on **(D)** from independent experiments. Images were analyzed in ImageJ. After background was subtracted, image was inverted and integrated density of each band was measured. Values were normalized to the integrated density from the corresponding lanes. Data presented in relation to a band intensity for GAPDH measured in each experiment. **(F)** Total cellular levels of gp91phox in untreated (Control) and exposed to swollen conidia for 2 h macrophages. A relative CTCF was calculated in relation to an average of CTCF measured in untreated (Control) BMDMs. **(G)** Uptake of swollen conidia by BMDMs was calculated by counting an average number of conidia per macrophage and was expressed as a percentage of the initial inoculum where MOI was set to 100%. Data are from two **(B, C, F)**, or three **(E, G)** independent experiments. “n” indicates number of phagosomes **(B)** or cells **(C, F, G)** used for quantification; bars indicate means and standard errors. Statistical significance was calculated with the one-way ANOVA **(B, G)**, Kruskal-Wallis test followed by Tukey post-hoc test **(C)**, or *t*-test **(E, F)**: * indicates p<0.05, ** indicates p<0.01, and *** indicates p<0.001.

It was suggested previously that NADPH oxidases could affect mitochondrial function and particularly mitoROS production, and *vice versa* (47, 48). Also, the infection-associated metabolic reprogramming of macrophages requires the activity of the NOX2 complex (10). However, it is not known whether NOX2 (gp91phox) activity is required to initiate RET-associated mitoROS generation in activated immune cells. Therefore, we tested whether infecting macrophages with swollen conidia induces a simultaneous increase in NOX2 phagosomal accumulation and mitoROS production. Interestingly, during first hour of co-incubation of BMDMs with swollen conidia, there was enhanced recruitment of NOX2 to phagosomal membranes (**Figures 5A, B**). During this period mitoROS was not elevated in infected cells, but mitoROS generation did follow after 1.5 h of infection (**Figure 5C**). Therefore, NOX2 activation precedes enhanced mitoROS production. We then tested whether the increase in mitoROS is associated with changes in NOX2 abundance rather than its recruitment to the phagosome. However, both western blot and microscopic analyses revealed no detectable alterations in NOX2 protein abundance after 2 h of infection, during which time mitoROS levels were increased (**Figures 5D–F**). Therefore, enhanced NOX2 phagosomal recruitment precedes, and might be required for, the elevation of mitoROS generation. Interestingly, the induction of the elevated mitoROS generation were not depended on the uptake of swollen conidia as the majority of conidia were engulfed during the first 0.5 h of infection (**Figure 5G**).

**Figure 6:**
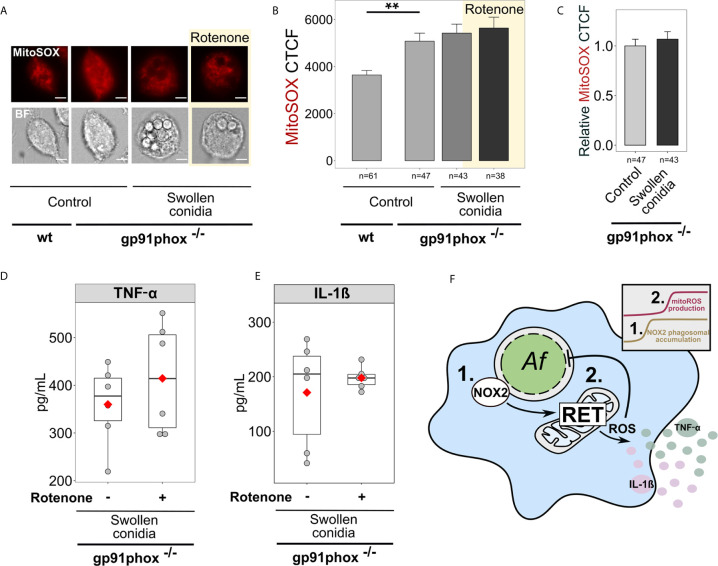
Regulation of cytokine secretion by mitoROS relies on the presence of NOX2 (gp91phox). **(A)** Microscopic analysis of mitoROS in BMDMs from wild-type (wt) or NOX2-deficient (gp91phox^-/-^) mice. Cells were either left untreated (Control) or infected with swollen *A. fumigatus* conidia for 2 h, stained with mitoSOX, fixed and subjected to imaging. Where indicated, BMDMs were pre-treated with rotenone (shown with yellow background) for 1 h. The scale bars are 5 µm. **(B)** The abundance of mitoROS in BMDMs was determined by image analysis of MitoSOX-stained BMDMs with ImageJ software. Means of CTCF for each condition were compared. **(C)** MitoSOX CTCF was measured in untreated (Control) gp91phox^-/-^ BMDMs and those exposed to swollen conidia for 2 h. A relative CTCF was calculated in relation to an average of CTCF measured in untreated (Control) gp91phox^-/-^ BMDMs. **(D, E)** Evaluation of cytokine secretion by infected NOX2-deficient (gp91phox^-/-^) BMDMs. Macrophages were infected with swollen conidia and supernatants were collected after incubation overnight. Levels of TNF-α and IL-1β in supernatants were analyzed by ELISA. No cytokines were detected in the supernatant from BMDMs (gp91phox^-/-^) treated rotenone, but not treated with swollen conidia (not shown). **(F)** Schematic of the regulation of antifungal responses in macrophages through NOX2 complex and RET-associated mitoROS production. 1. Following infection with swollen conidia, gp91phox is recruited to the phagosomal membrane. 2. MitoROS production is increased due to RET. Activation of gp91phox is necessary for the regulation on cytokine secretion *via* RET-associated mitoROS. Data are from two independent experiments. “n” indicates number of cells used for quantification; bars indicate means and standard errors, red rhomb represent means. Statistical significance was calculated with the Kruskal-Wallis test followed by Tukey post-hoc test **(B)**, the Mann Whitney U Test **(C)**, the *t*-test **(D, E)**: ** indicates p<0.01.

Remarkably, NOX2 deficient BMDMs (gp91phox^-/-^) exhibited higher basal levels of mitoROS in a resting state compared to wild-type BMDMs (**Figures 6A, B**). However, following challenge with swollen conidia, gp91phox^-/-^ macrophages displayed no significant increase in mitoSOX cellular fluorescence (**Figures 6A–C**). Also, rotenone did not influence mitoROS levels in stimulated NOX2-deficient cells (**Figures 6A, B**). Accordingly, inhibition of RET with rotenone did not affect cytokine secretion in stimulated gp91phox^-/-^ BMDMs, suggesting that RET did not occur in those cells (**Figures 6D, E**).

To examine the role of NADPH oxidase and associated mitoROS in inhibition of *A. fumigatus* growth by immune cells, we treated macrophages with diphenyleneiodonium (DPI), an NADPH oxidase inhibitor (49). First, we confirmed that as in NOX2-deficient cells, cytokine levels were not significantly affected by rotenone in DPI-treated BMDMs exposed to swollen conidia (**Supplementary Figure 2A**). Furthermore, the inhibitors of RET-mediated mitoROS, rotenone and S1QEL1.1, did not change the fungicidal activity of DPI-treated macrophages infected either with resting or swollen conidia (**Supplementary Figures 2B, C**). This provided additional evidence that RET did not take place in infected cells with the defective NADPH oxidase. Also, the observed impact of FCCP, which disrupts mitochondrial membrane potential, suggested that mitochondria might be depolarized without a functional NADPH oxidase. Evidently, additional depolarization caused by FCCP negatively affected the fungicidal activity of BMDMs (**Supplementary Figures 2B, C**). To further examine the importance of NADPH oxidase and downstream mitoROS signalling *via* RET, we prolonged incubation of BMDMs with swollen conidia. Then, we discovered that after 6 h of infection, about 14% of fungal growth was inhibited by control macrophages, while only 7% of fungal growth was inhibited by DPI-treated macrophages, which was consistent with the previous report on alveolar macrophages (50). This suggested that while untreated macrophages could stop fungal proliferation, macrophages with the inhibited NADPH oxidase failed to prevent *A. fumigatus* growth. This confirmed the role of NADPH oxidase in sustained fungal growth inhibition by macrophages.

In summary, NOX2 defects prevent RET-associated mitoROS production and disrupt anti-*Aspergillus* functions of macrophages mediated *via* this mechanism. This reveals a link between NOX2 and mitoROS production (**Figure 6F**) that is required for the regulation of antifungal responses in macrophages.

## Discussion

This study addresses how mitoROS levels in macrophages orchestrate antimicrobial responses against the major fungal pathogen *A. fumigatus*. We found that increased mitoROS levels are triggered by germinating (swollen) conidia of *A. fumigatus*, but not by dormant (resting) conidia. RET contributes to these mitoROS levels, and this is related to elevated complex II activity in the respiratory chain and increased mitochondrial membrane potential. The elevation in mitoROS levels appears to rely on upstream NOX2-dependent signalling, ultimately leading to the enhanced production of pro-inflammatory cytokines that include TNF-α and IL-1β. Together, these results reveal that RET-generated mitoROS contribute to the antifungal responses of macrophages to *A. fumigatus*.

Coenzyme Q binding sites at complex I and complex III are among major producers of ROS in mitochondria (51). MitoROS generated at complex III was proposed to regulate cytokine secretion in *Listeria monocytogenes*–infected macrophages (52). Interestingly, in our study, a compound that specifically inhibits ROS production at coenzyme Q binding site of complex III, namely S3QEL2 (53), did not alter the ability of macrophages to inhibit *A. fumigatus* growth or secrete TNF-α. This suggested that complex III was not the main source of elevated mitoROS in fungus-stimulated macrophages.

It has been also proposed that enhanced mitoROS production *via* RET at complex I is crucial to transform macrophages into a pro-inflammatory state upon activation with LPS (9). The process involves metabolic alterations in the macrophage that include increased succinate oxidation by complex II, which could potentially drive mitoROS production *via* RET (9, 10). Here, we demonstrate that exposure to swollen *A. fumigatus* conidia leads to elevated complex II activity in the infected macrophages, and to mitoROS formation *via* RET. Interestingly, fungal viability is not essential to trigger RET, as formalin-fixed swollen conidia were sufficient to initiate mitoROS *via* this pathway. However, given that heat-killed swollen conidia fail to induce elevated mitoROS levels, heat-sensitive molecules on the fungal surface, or the heat-sensitive organization of these molecules on the surface, seem to trigger RET in the macrophage.

One of the phenotypic outcomes of enhanced mitoROS in stimulated macrophages is the modulation of cytokine release. The exact mechanism by which mitoROS affect cytokine secretion remains unclear. However, we have shown that macrophages infected with swollen *A. fumigatus* conidia exhibit increased thiol oxidation in endogenous proteins. This is consistent with the report that in *L. monocytogenes*-infected macrophages, mitoROS modify the redox status of Nuclear factor-κB (NF-κB) essential modulator, which regulates the extracellular signal-regulated protein kinases 1 and 2 and NF-κB pathways to promote the synthesis of pro-inflammatory molecules such as IL1-β, TNF-α and IL-6 (52). In addition to regulating redox-sensitive regulators of cytokine secretion, mitoROS may also affect pathways that supply reducing equivalents, such as NADPH. For instance, mitoROS can cause reversible oxidative modifications of glyceraldehyde 3-phosphate dehydrogenase (GAPDH), which would result in the diversion of glycolytic intermediates towards the pentose phosphate pathway to generate NADPH (54, 55). In addition to providing the reducing power that fuels antioxidant systems (56), NADPH is also used by NOX2 to generate antimicrobial phagosomal ROS. The potential involvement of mitoROS in regulating GAPDH activity, and hence the supply of reducing equivalents for NOX2, requires further investigation.

In addition to the potential impact of mitoROS on NOX2 functionality, NOX2 itself directly influences mitoROS production in activated macrophages. This work confirms there is crosstalk between NOX2 and mitochondria. In particular, using NOX2-deficient cells, we have shown that NOX2 activity is required to trigger mitoROS through RET. It is conceivable that, upon infection, NOX2-derived ROS cause oxidation and activation of the Fgr kinase leading to enhanced mitochondrial complex II activity, thereby creating the conditions for RET to take place (10, 57). Alternatively, the ROS produced by NOX2 may cause depolarization of mitochondrial membranes, matrix swelling and alkalinization, thereby leading to elevated mitoROS levels (58–60). Interestingly, the inhibition of NOX2 complex assembly in the phagosome membrane by DHN melanin on the conidial surface (61) could have accounted for the lack of mitoROS production by macrophages exposed to resting conidia. However, this notion is not supported by our observation that dormant Δ*pksp* conidia, which lack DHN melanin, were unable to induce RET in macrophages. Taken together, these findings indicate that NOX2 activation, in conjunction with the recognition of fungal cell surface components unique to swollen conidia, are required to initiate RET-mediated mitoROS redox signalling.

The lack of RET-mediated mitoROS in macrophages after exposure to resting conidia may explain their limited inflammatory responses and their ability to preserve immune homeostasis (62). Upon inhalation of *A. fumigatus* conidia, pulmonary immune cells maintain sterilizing immunity by rapid clearance of these resting conidia (63). The absence of mitoROS signalling may help to prevent exaggerated immune responses to dormant conidia that do not pose a significant threat. On the other hand, we observed an increase in RET-mediated mitoROS production following the sensing of a more dangerous form of the fungus - germinating conidia with the potential to cause invasive disease. Therefore, the regulation of mitoROS production *via* RET may be important for scaling immune responsiveness by activating immune responses to swollen *A. fumigatus* while preventing inflammation to dormant conidia.

As well as being important for initiating pro-inflammatory signals, RET in immune cells might be involved in limiting inflammation and preventing tissue damage after prolonged stimulation (11, 12). Thus, it is intriguing to speculate that in NOX2-deficient cells, the absence of RET might be responsible for dysregulated inflammatory responses. Indeed, patients with chronic granulomatous disease (CGD) whose immune cells lack functional NOX2, suffer from prolonged inflammatory reactions that can lead to tissue damage (64, 65). Although RET-mediated mitoROS production does not occur in NOX2-deficient cells, and thus does not contribute into cytokine induction, our data indicate that redox homeostasis might be disrupted in these cells due to high mitoROS levels. This would be consistent with previous studies claiming that the lack of NOX2 results in mitochondrial redox imbalance and oxidative stress (66–68). In particular, in CGD patients, increased mitoROS levels are responsible for spontaneous neutrophil extracellular trap formation and linked to autoimmune disorders (67). Therefore, further investigations are required to address whether the activation of RET in immune cells with redox imbalance, such as gp91phox^-/-^ cells, would help to modulate and normalize dysregulated inflammatory reactions. To support this possibility, triggering RET was previously shown to rescue pathogenesis associated with severe oxidative stress and even to increase *Drosophila* lifespan (18). Therefore, the modulation of mitoROS production specifically *via* RET might be beneficial in various pathological conditions.

## Data Availability Statement

The raw data supporting the conclusions of this article will be made available by the authors, without undue reservation.

## Ethics Statement

The animal study was reviewed and approved by UK Home Office (project license P79B6F297).

## Author Contributions

All authors contributed to the article and approved the submitted version.

## Funding

The work was funded by the Medical Research Council Centre for Medical Mycology at the University of Exeter [MR/N006364/2]. GB is funded by the Wellcome Trust, and AB by the Medical Research Council [MR/M026663/2]. The funders had no role in study design, data collection and analysis, decision to publish, or preparation of the manuscript.

## Conflict of Interest

The authors declare that the research was conducted in the absence of any commercial or financial relationships that could be construed as a potential conflict of interest.

## Abbreviations

MitoSOX, red mitochondrial superoxide indicator; mitoROS, mitochondrial ROS; RET, reverse electron transport; NADPH, nicotinamide adenine dinucleotide phosphate; NOX, NADPH oxidase; ELISA, enzyme-linked immunosorbent assay; gp91phox^-/-^, NOX2 deficiency; ROS, reactive oxygen species; CGD, chronic granulomatous disease; LPS, lipopolysaccharide; IL-1β, interleukin-1β; TNF-α, tumor necrosis factor-α; CTCF, corrected total cell fluorescence; BMDM, bone marrow-derived macrophages; MOI, multiplicity of infection; GLRX1, glutaredoxin 1; SOD2, mitochondrial superoxide dismutase; GAPDH, glyceraldehyde 3-phosphate dehydrogenase; DHN, 1,8-dihydroxynaphthalene; CI, complex I; CII, complex II; CS, citrate synthase; NF-κB, nuclear factor-κB; DPI, diphenyleneiodonium chloride.
