# Supplementary material for: Mitochondrial Reactive Oxygen Species Regulate Immune Responses of Macrophages to Aspergillus fumigatus
Source: Front Immunol. 2021 Mar 25;12:641495. doi: 10.3389/fimmu.2021.641495 (PMC8026890; doi:10.3389/fimmu.2021.641495)
Supplement: Supplementary file 1 [file DataSheet_1.docx]

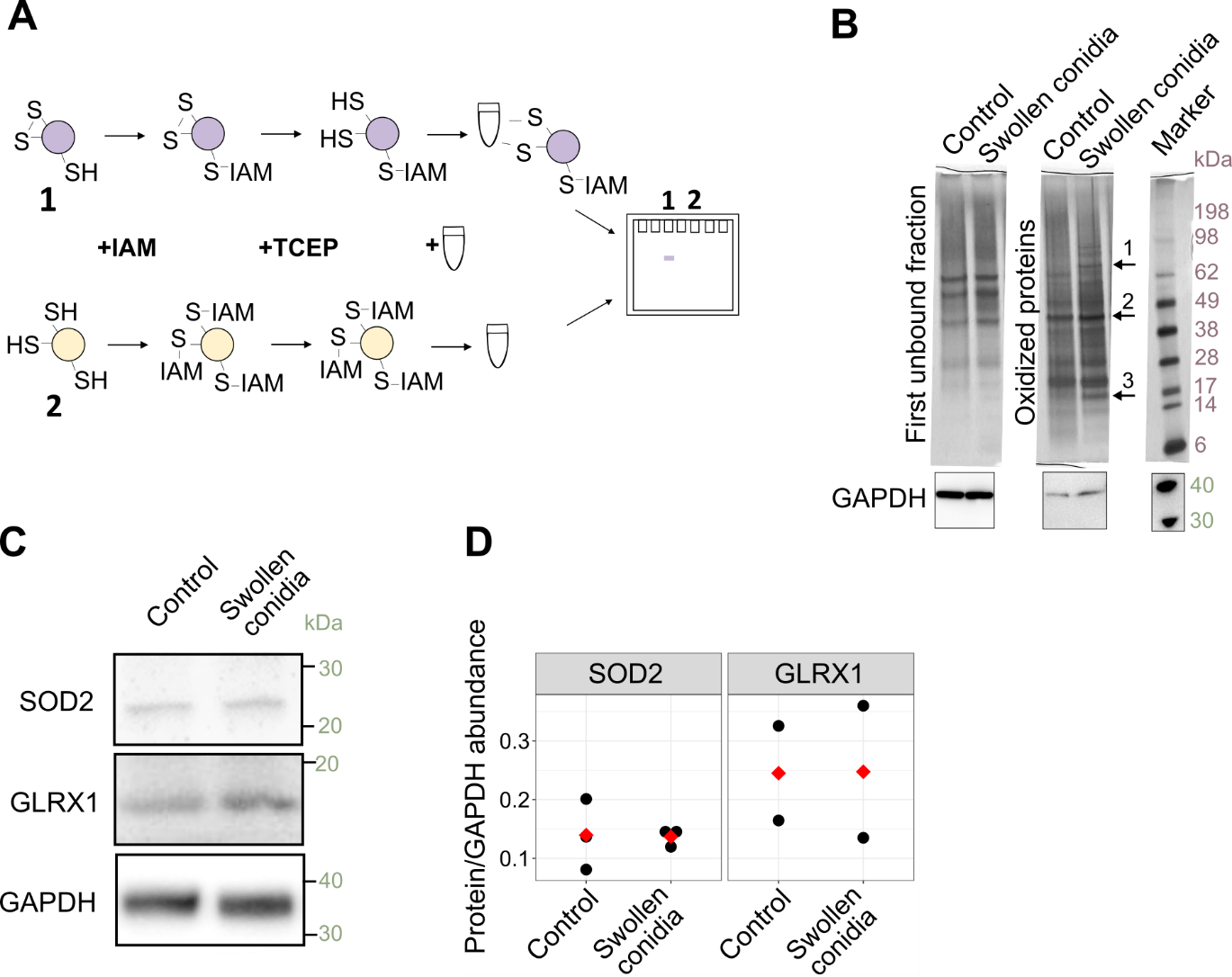


**FigSup1**: Swollen conidia of *A. fumigatus* induce increased levels of intracellular reversibly oxidized proteins in BMDMs, but infected cells do not exhibit oxidative stress.

**(A)** Schematic diagram of gel-based strategy for analysis of reversible oxidative cysteine modifications. Free thiol groups (-SH) are blocked by alkylation with iodoacetamide (**IAM**) and different types of oxidatively modified cysteines such as disulphides reduced by tris(2-carboxyethyl)phosphine (**TCEP**). Next, free thiols originated from modified cysteines are bound to thiopropyl Sepharose resin allowing to capture only oxidised proteins (**1**) while leaving reduced proteins (**2**) in unbound fractions. After removing the unbound fractions, enriched proteins are eluted, separated by gel electrophoresis, and visualized by protein staining. **(B)** SDS gel and western blot images of enriched oxidized proteins from BMDMs infected with swollen conidia of *A. fumigatus.* BMDMs were incubated with *A. fumigatus* swollen conidia for 2 h, proteins were isolated, pre-processed and subjected to thiol-affinity enrichment. The unbound fraction eluted from the resin represents unmodified proteins and proteins that do not contain cysteines. Enriched proteins from resting (Control) and infected (Swollen conidia) BMDMs are shown as oxidized proteins. Arrows indicate selected bands, which show increased intensity when compared in resting and infected BMDMs. Both unbound and enriched fractions of proteins were analysed by western blot with anti-GAPDH antibodies. **(C)** Western blot analysis of protein abundance in resting (Control) and infected for 2 h with swollen conidia BMDMs. Proteins were extracted from macrophages and analysed by western blot with anti-GLRX1, SOD2, or GAPDH antibodies. **(D)** Quantification of intensity of bands on blots shown on C) from independent experiments. Images were analysed in ImageJ. After background was subtracted, image was inverted and integrated density of each band was measured. Values were normalized to the integrated density from the corresponding lanes. Data presented in relation to a band intensity for GAPDH measured in each experiment.


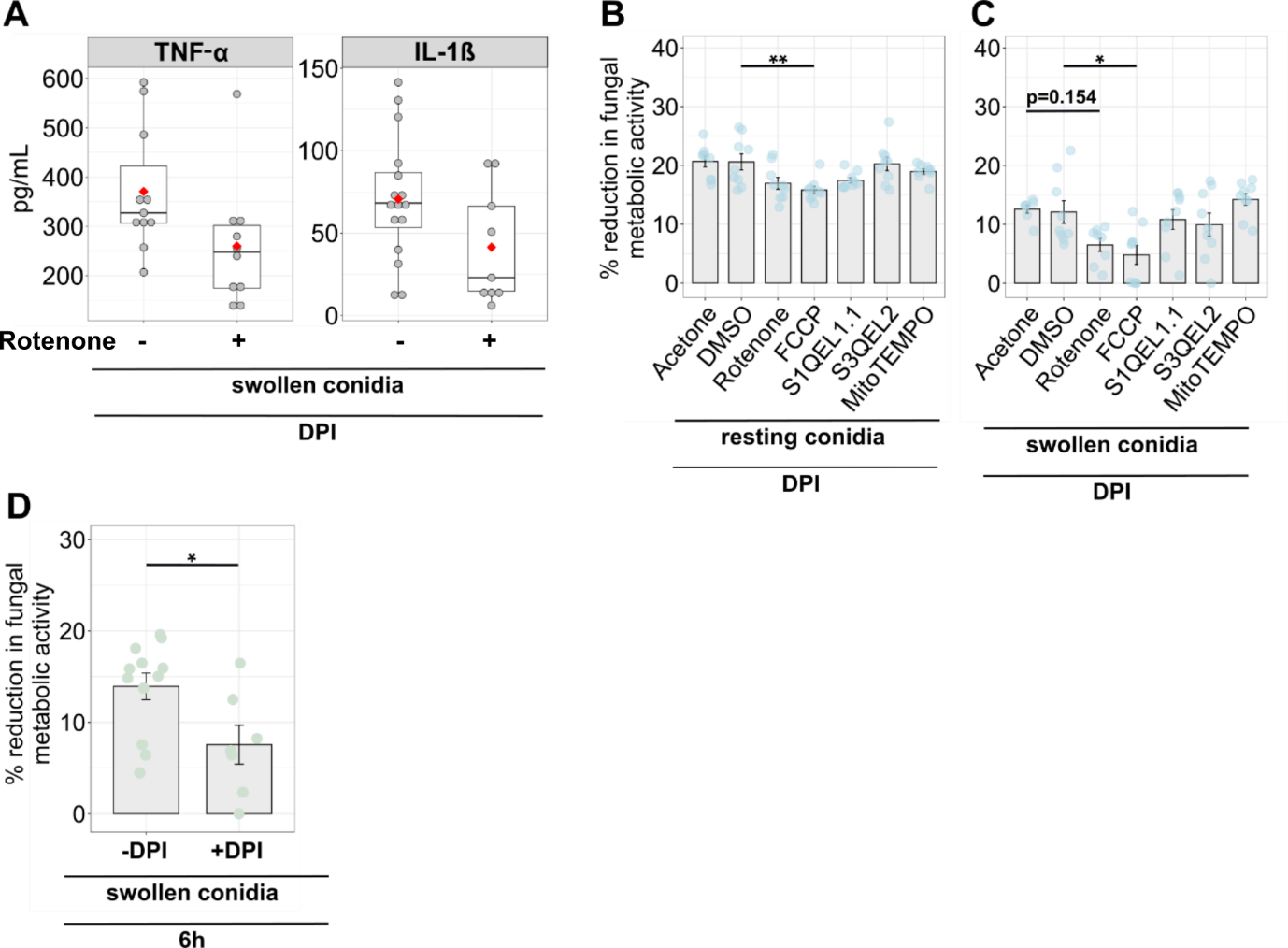


**FigSup2**: Regulation of cytokine secretion and fungicidal function by mitoROS relies on the activity of NADPH oxidase.

**(A)** Evaluation of cytokine secretion by infected DPI-treated macrophages. Bone marrow-derived macrophages (BMDMs) were first incubated with DPI for 1 h, then cells were treated either with vehicle (acetone) or rotenone for 1 h, after washing, macrophages were exposed to swollen *A. fumigatus* conidia and the supernatant was collected after incubation overnight. Levels of TNF-α (A) and IL-1β (B) in supernatants were analysed by ELISA. **(B, C)** BMDMs were first incubated with DPI for 1 h, which followed by treatment with vehicle (acetone or DMSO), rotenone, or FCCP for 1 h. After compounds were removed, BMDMs were treated with swollen or resting *A. fumigatus* conidia. Antioxidants S1QEL1.1, S3QEL2, or mitoTEMPO were added together with conidia where indicated. After incubation for 4 h, macrophages were lysed with water containing Triton X-100, and the growth of *A. fumigatus* was measure by a metabolic activity assay based on resazurin. The metabolic activity of *A. fumigatus* conidia that were not exposed to BMDMs was set to 100%. **(D)** BMDMs were treated either with vehicle (DMSO) or DPI for 1 h. After washing, BMDMs were exposed to swollen *A. fumigatus* conidia. After incubation for 6 h, macrophages were lysed with water containing Triton X-100, and the growth of *A. fumigatus* was measure by a metabolic activity assay based on resazurin. The metabolic activity of *A. fumigatus* conidia that were not exposed to BMDMs was set to 100%. Data are from two (A), or three (B, C, D) independent experiments. Bars indicate means and standard errors, red rhomb represent means. Statistical significance was calculated with the Student’s *t*-test (A, D), or one-way ANOVA (B, C) followed by Tukey post-hoc tests: * indicates p<0.05, ** indicates p<0.01.
